# Treatment strategies for rapid recurrence and progression after surgery for stage I pulmonary pleomorphic carcinoma: a case report and literature review

**DOI:** 10.3389/fimmu.2025.1547872

**Published:** 2025-06-04

**Authors:** Jing Zhang, Wenji Xiong, Hong Wang, Lemeng Sun, Chen Chen, Xueru Sun, Xiuyue Man, Lei Yang, Zhihong Li

**Affiliations:** ^1^ Department of Cancer Center, The First Hospital of Jilin University, Changchun, Jilin, China; ^2^ Department of Radiology, The First Hospital of Jilin University, Changchun, Jilin, China; ^3^ Department of Thoracic Surgery, The First Hospital of Jilin University, Changchun, Jilin, China

**Keywords:** pulmonary pleomorphic carcinoma, sintilimab, immune checkpoint inhibitors, immunotherapy, non-small cell lung cancer

## Abstract

Pulmonary pleomorphic carcinoma (PPC) is a rare and poorly differentiated subtype of non-small cell lung cancer (NSCLC), primarily originating from non-epithelial tissue. Currently, there is no consensus on optimal treatment strategies for patients with PPC presenting diverse clinical features. This report describes an elderly male with stage I pulmonary pleomorphic carcinoma. Five months after surgery, the disease progressed rapidly, resulting in vertebral metastasis. Following a second surgery, the patient was treated with a programmed cell death protein-1 (PD-1) inhibitor combined with cytotoxic therapy. This regimen led to significant tumor shrinkage, achieving a partial response (PR). These findings suggest that combining immune checkpoint inhibitors (ICIs) with chemotherapy may improve outcomes for patients with advanced PPC.

## 1 Introduction

PPC is an aggressive subtype of NSCLC classified under pulmonary sarcomatoid cancers, accounting for less than 1% of all lung cancer cases (1). This cancer demonstrates resistance to traditional chemotherapy and radiotherapy, is associated with a poor prognosis, and exhibits rapid progression in advanced stages. According to previous studies, the 5-year survival rate for PPC patients is approximately 23%, with a median overall survival of 9 months (2). Compared to other NSCLC subtypes, PPC shows higher PD-L1 expression and greater immune cell infiltration (3). Currently, no standard treatment protocol exists for PPC. Surgical resection is considered the most effective approach for early-stage PPC and plays a key role in reducing recurrence and metastasis. However, postoperative recurrence and survival rates are influenced by multiple factors; for example, PPC with adenocarcinoma components has been associated with higher recurrence rates (4). This report highlights the case of a patient with early-stage PPC containing an adenocarcinoma component, who experienced rapid postoperative progression. Remarkably, significant clinical improvement was achieved following the administration of the PD-1 inhibitor sintilimab combined with cytotoxic drugs and therapies targeting vascular and bone metastasis. The clinical and pathological features of this patient were analyzed to provide a novel treatment reference for managing advanced PPC.

## 2 Case presentation

**Figure 1:**
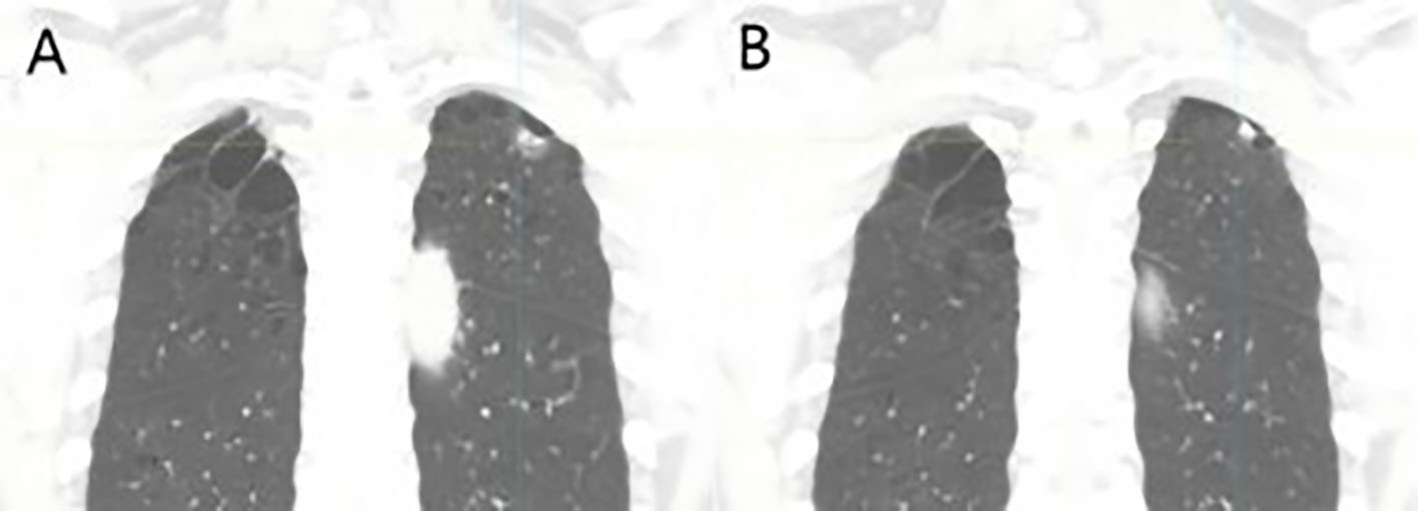
Coronal reconstructed images at initial diagnosis: **(A)** A subsolid nodule located on the parietal pleura in the apical segment of the left upper lobe, measuring approximately 15 × 10 mm, and **(B)** a solid component within the nodule, approximately 7 mm in diameter.

**Figure 2:**
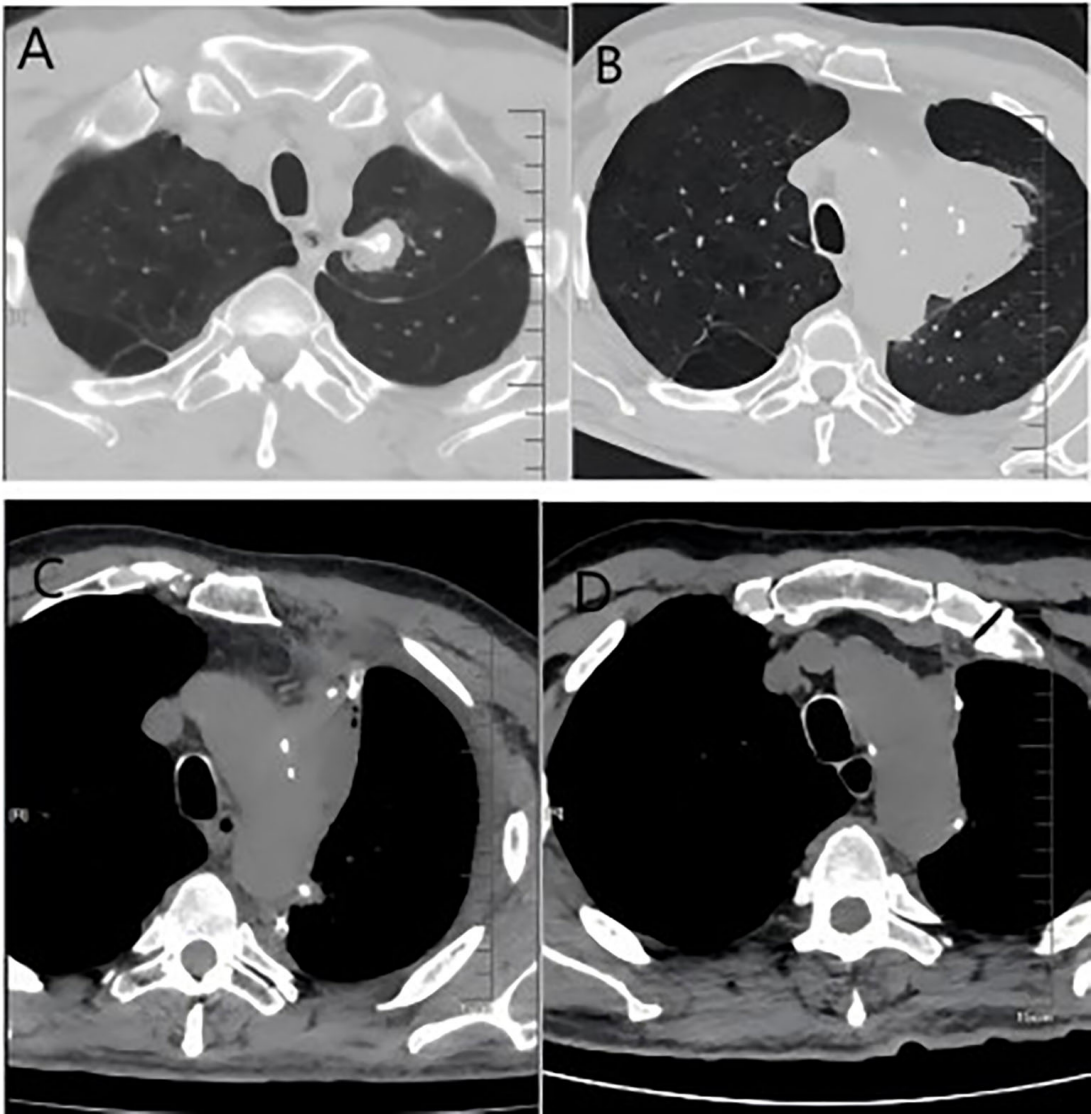
**(A)** Imaging from the first follow-up after the initial surgery showing post-surgical scarring; **(B)** imaging at recurrence, demonstrating a new soft tissue mass in the operated area; **(C)** Images after the second surgery; **(D)** Images after 6 courses of systemic therapy.

**Figure 3:**
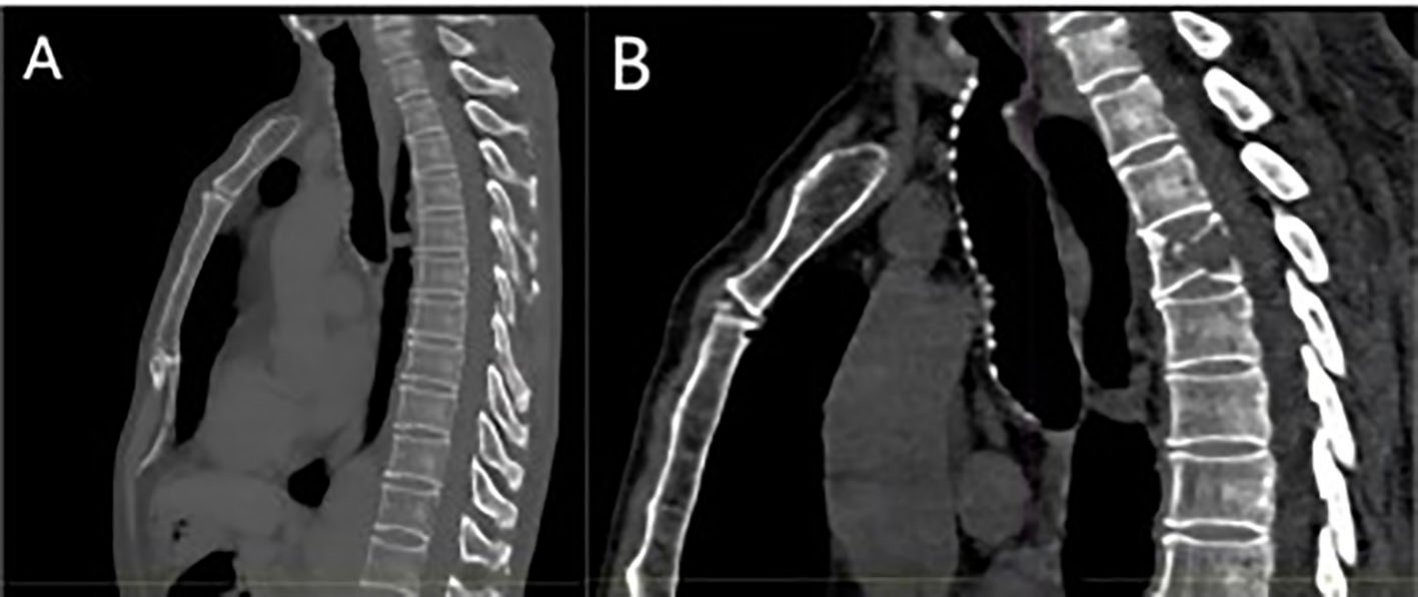
**(A)** Baseline bone window examination showing no evidence of destructive bone changes; **(B)** imaging at recurrence demonstrating hypodense bone destruction and a fracture of the third lumbar vertebra.

The patient, a 67-year-old male, presented to the Department of Thoracic Surgery in November 2023 for evaluation following a physical examination that detected a space-occupying lesion in the upper lobe of the left lung. The lesion was a subsolid nodule located adjacent to the pleura of the apical segment of the upper lobe of the left lung, measuring approximately 15 x 10 mm and containing a solid nodule approximately 7 mm in diameter (**Figure 1**). He had a smoking history of approximately 20 pack-years but no abnormalities in tumor markers including CEA, CYFRA21-1, SCCA, NSE. The patient had been healthy, with no specific family history of malignancy. His performance status was European Cooperative Oncology Group (ECOG) 0. The patient underwent a comprehensive examination, including computed tomography(CT) scans of the head, chest, and abdomen, an ultrasound of the superficial lymph nodes, and a whole-body bone scan. Based on clinical findings, the diagnosis was stage IA occupational lesion of the left lung. The patient underwent intrinsic upper lobe resection of the left lung. Intraoperative frozen-section histopathology revealed microinvasive adenocarcinoma and negative margins, so total lobectomy was not performed. Postoperative pathology identified pleomorphic carcinoma, measuring 1.3 cm. The tumor consisted of approximately 50% spindle cell carcinoma and 50% hyperdifferentiated adenocarcinoma. Immunohistochemistry results included Ki-67 (+40%), Napsin A (partly +), TTF-1 (+), CK7 (+), CK5/6 (-), P40 (-), and CKpan (+). The final diagnosis was stage IA (pT1N0M0) pleomorphic carcinoma of the left lung. The comprehensive genetic sequencing revealed no abnormal gene mutations in the patient. During the first postoperative follow-up in April 2024 (progression-free survival [PFS] = 5 months), the patient presented with a cough and hemoptysis. Lung computed tomography (CT) revealed a mass at the surgical site (**Figure 2B**) and metastases in the thoracic third vertebral body (**Figure 3**), leading to a diagnosis of stage IV recurrent pleomorphic carcinoma of the left lung with bone metastases. The patient’s head magnetic resonance imaging (MRI) and abdominal CT scan examination showed no metastases to other sites.

**Figure 4:**
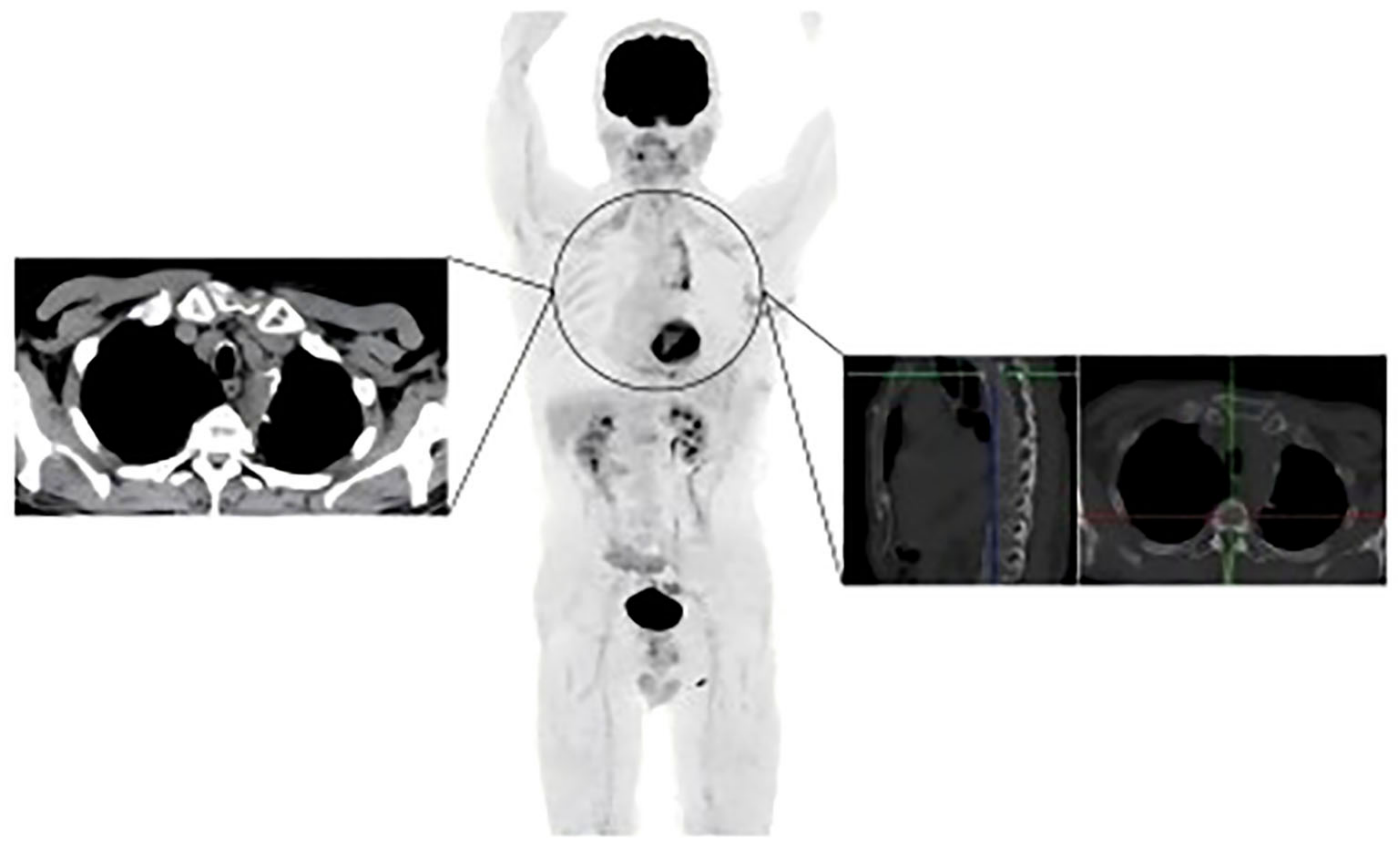
The PET-CT scan results for the patient from December 2024.

**Figure 5:**
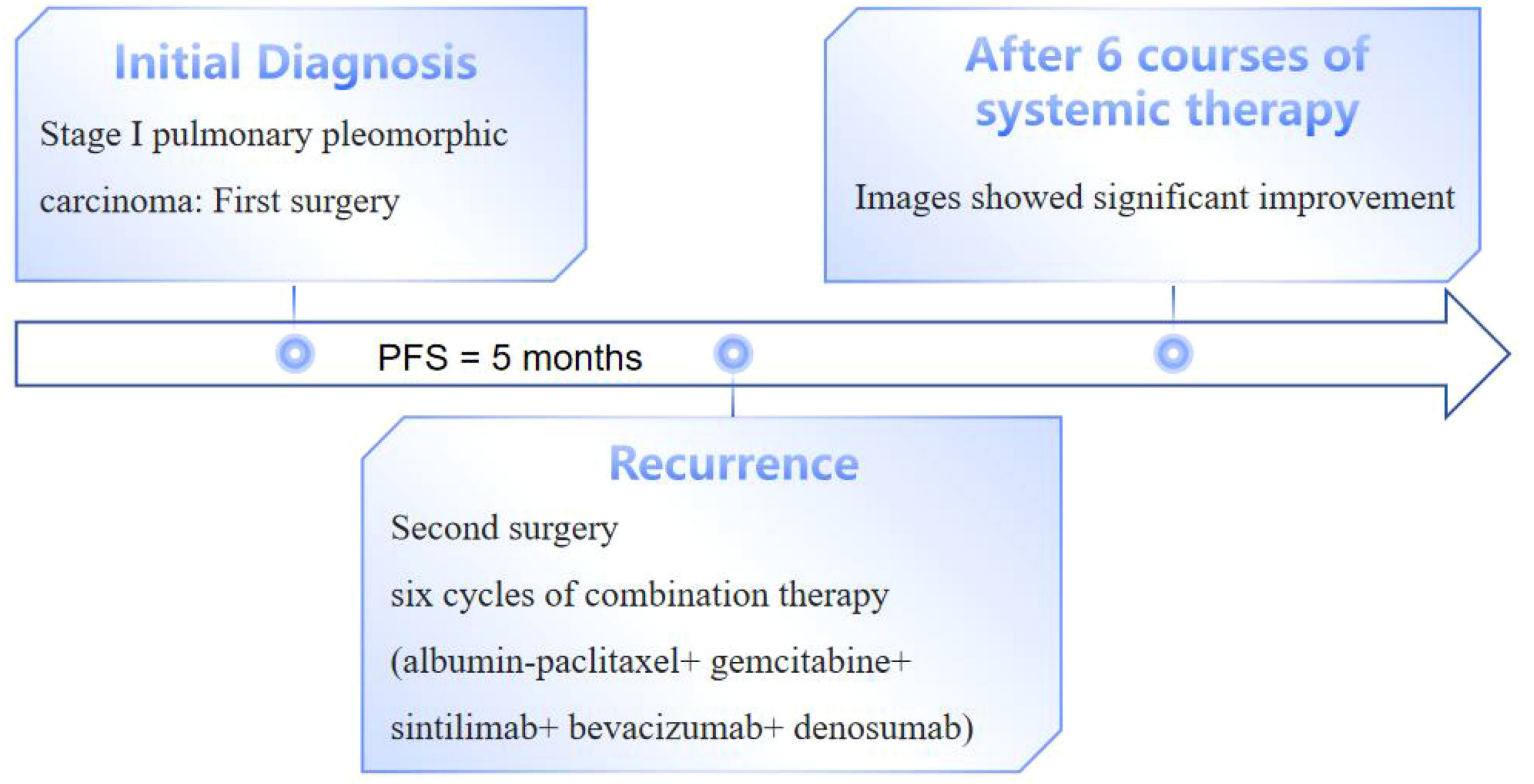
Clinical course of the patient in this case.

In May 2024, the patient underwent resection of the lingual segment of the left upper lobe (**Figure 2C**). Pathology confirmed pleomorphic carcinoma, with tumor composition of 50% spindle cell carcinoma, 30% giant cell carcinoma, and 20% adenocarcinoma. The tumor measured 2.5 cm × 2 cm × 1 cm. Immunohistochemistry results included Ki-67 (+60%), CKpan (+), CK7 (partly +), Napsin A (partly +), TTF-1 (partly +), P40 (-), P63 (-), vimentin (+), SMA (partly +), desmin (-), SMARCA4 (+), INI-1 (+), and PD-L1 (SP263; tumor proportion score [TPS]: 80%). Postoperatively, the patient received six cycles of combination therapy, including albumin-paclitaxel, gemcitabine, sintilimab, bevacizumab, and denosumab. The treatment consistently achieved a partial response (PR) based on efficacy evaluations (**Figure 2D**). In December 2024, PET-CT scanning showed that the patient had achieved PR in the lesion, suggesting that the treatment regimen was effective (**Figure 4**). The clinical course of the patient is illustrated in **Figure 5**. As of February 2025, the patient is continuing with 6 cycles of treatment using sintilimab and bevacizumab, achieving a progression-free survival of up to 9 months.

## 3 Discussion and literature review

Studies have indicated that PPC is more prevalent in older adults, with a median age range of 60–70 years. It is more common in men, with a male-to-female ratio of approximately 1.40:1, and is strongly associated with smoking. Across racial groups, about 80% of patients with PPC are Caucasian (2). Clinical symptoms of PPC can include chest pain, a persistent cough, hemoptysis, dyspnea, fever, and weight loss (5), though rare manifestations such as glandular cystitis and secretion of βHCG have also been reported (6). In this case, the patient, a 67-year-old male with a history of smoking, presented with a space-occupying lesion in the upper lobe of the left lung detected during a physical examination. The patient reported occasional dry cough and a weight loss of 4 kg. No other significant clinical symptoms or abnormal test results were observed, likely due to the tumor’s initial location and microscopic nature.

On chest computed tomography (CT), PPC lesions are typically peripheral, located in the upper lobes, with smooth margins and large sizes. They often show internal necrosis, unevenly thickened margins on enhanced scans, and “ice-floating” changes. Peritumoral ground-glass opacities are frequently observed, along with pleural invasion, hilar involvement, and mediastinal lymph node metastases (7). Studies suggest that lesions in the lobes have a better prognosis than those in the main bronchus, and patients with right lung lesions may have better overall survival (OS) than those with left lung lesions (2). At initial diagnosis, this patient’s CT revealed a lesion in the upper lobe of the left lung measuring approximately 1.6 cm × 1.1 cm. The lesion displayed ground-glass opacities with a solid nodule component pulling on the adjacent pleura. The imaging findings differed from those typically seen in larger lesions, likely due to the tumor being detected at an earlier stage.

**Figure 6:**
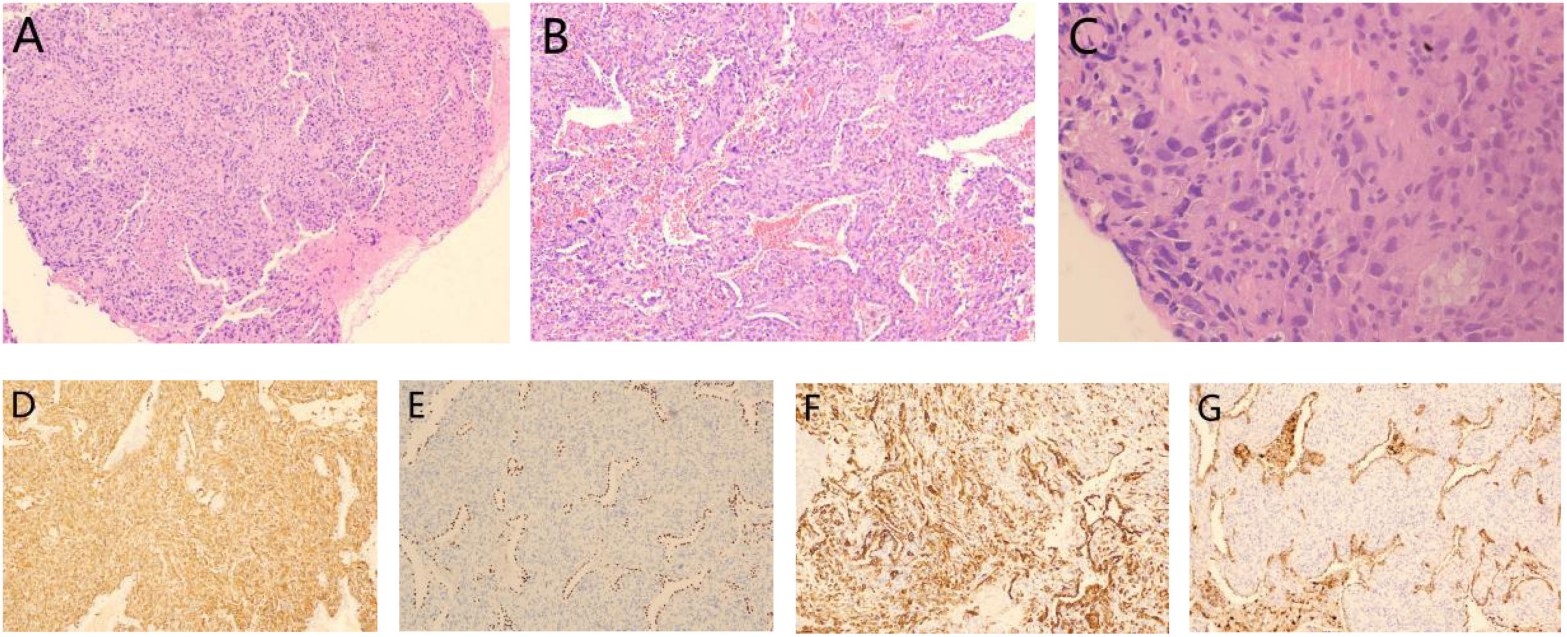
Pathological results from the second surgery: **(A)** Mixed presence of giant cells and spindle cells: HE staining(x40); **(B)** Adenocarcinoma components: HE staining(x100) and **(C)** Giant cells in tumor tissue: HE staining(x400). Immunohistochemistry results showed **(D)** vimentin (+), **(E)** TTF-1 (+), **(F)** CK7 (+) and **(G)** Napsin A (+).

Pathologically, pleomorphic carcinoma, a subtype of sarcomatoid carcinoma, is defined by the presence of at least 10% spindle cell or giant cell components, or by tumors entirely composed of spindle cells or neoplastic giant cells (8, 9). The mixed subtype of PPC has a better prognosis than the pure subtype (10). In this case, the initial tumor consisted of approximately 50% spindle cell carcinoma and 50% hyperdifferentiated adenocarcinoma. After recurrence, the tumor pathology showed 50% spindle cell carcinoma, 30% giant cell carcinoma, and 20% adenocarcinoma, indicating increased tumor heterogeneity. The higher proportions of spindle cell and giant cell components raise questions about their potential impact on prognosis, warranting further investigation. And in this patient (**Figure 6**), immunohistochemistry revealed positive expression of CK7, Napsin A, TTF-1, and vimentin, consistent with the immunohistochemical profiles of most pleomorphic carcinomas (7).

Previous studies have shown that adjuvant chemotherapy reduces the risk of death by 46% in patients with distant metastases but does not significantly improve survival in patients with early-stage or locally advanced disease (2). In this case, the patient was initially diagnosed with stage IA and did not receive adjuvant therapy after surgery. However, the disease recurred within 5 months, suggesting that adjuvant therapy may be necessary even for stage IA patients to reduce the risk of recurrence. Following the second surgery, the patient was treated with a combination regimen of albumin-paclitaxel, gemcitabine, sintilimab, and bevacizumab, resulting in significant efficacy and a PR. Here we list some previously reported clinical cases of pleomorphic carcinoma of the lung (**Table 1**).

**Table 1:** Clinical cases of pulmonary pleomorphic carcinoma reported in the literature.

No.	Sex/ Age(y)	Stage	Treatment	Follow-up
1 Jiang, Tao et al.,2023 (36)	M/70	IV	surgical resection of the brain metastases, immunotherapy (pembrolizumab)	No progress in 13 cycles of immunotherapy
2 Jiang, Tao et al.,2023 (36)	M/60	I/II→IV	Radical surgery of the lung lobe → chemotherapy (pemetrexed/carboplatin) →immunotherapy (tislelizumab)	Recurrence 2 months after surgery →intolerant to chemotherapy, distant metastases 4 months later →no progress in 7 cycles of immunotherapy.
3 Zhang, Xiangwei et al.,2017 (37)	F/69	Ib	pneumonectomy	Living disease free for 12 months after the surgery
4 Yamasaki, Masahiro et al.,2021 (38)	M/78	IV	immunotherapy (pembrolizumab)	No progress in 8 months of immunotherapy
5 Kano, Yukari et al.,2022 (34)	F/43	IV	Osimertinib (EGFR-tyrosine kinase inhibitor)	No progress for 11 months
6 Dolkar, Tsering et al.,2024 (39)	M/40	IIb	chemotherapy with cisplatin/docetaxel, immunotherapy(atezolizumab)	No progress for 1 year
7 Matsuda, Masayuki et al.,2021 (40)	M/74	IIb→IV	Gastrojejunal bypass, followed by immunotherapy (pembrolizumab)	No treatment at first diagnosis. Duodenal metastases were found 1.5 years later.(OS=20 months)
8 Senoo, Satoru et al.,2019 (41)	M/62	IV	chemotherapy (pemetrexed/carboplatin) →immunotherapy(nivolumab)	First-line treatment: PFS=1 month; Second-line treatment: no progress for more than 1 year
9 Kim, Tae-Hun et al.,2021 (22)	M/80	IIIA	immunotherapy(pembrolizumab)+ intensity-modulated radiation therapy	No progress for 15 months

Regarding the choice of cytotoxic drugs, previous reports indicate that cytotoxic chemotherapy in cases of postoperative recurrence or unresectable PPC often leads to disease progression, with a median overall survival of approximately 5 months (11). However, a few patients with advanced PPC and distant metastases who were treated with gemcitabine or paclitaxel combined with platinum experienced recurrence-free survival exceeding 6 years (12–14). Based on this, a paclitaxel plus gemcitabine regimen was selected for this patient. For immunotherapy, PD-L1 expression is often positive in pleomorphic lung cancer, particularly in the sarcomatoid component (15, 16), and is associated with longer survival following immune checkpoint inhibitor (ICI) therapy (17). Given the patient’s high PD-L1 expression (tumor proportion score [TPS]: 80%), sintilimab, a PD-1 inhibitor, was administered.

PD-L1 expression in tumor cells and tumor-infiltrating lymphocytes (TILs) assessed by immunohistochemistry (IHC) is considered the most reliable predictor of response to immunotherapy in different tumors (18). There are two commonly used PD-L1 scoring methods for lung cancer: TPS (tumor proportion score)/TC (tumor cell score) and IC (immune cell score) (19). The Food and Drug Administration (FDA) approves the use of ICIs such as Pembrolizumab, Atezolizumab, and Durvalumab, provided that the PD-L1 assessment relies on a specific clone and scoring system (20). In contrast, the European Medicines Agency (EMA) permits treatment with ICIs without specific platforms or antibodies for immunohistochemistry testing.

Previous clinical reports suggest that patients with pleomorphic NSCLC may benefit from ICI treatment, but the efficacy can vary. For example, among patients treated with pembrolizumab alone or in combination with chemotherapy or radiation, some achieved progression-free survival of up to 15 months, while others showed no response (21–23). In three patients with high PD-L1-expressing PPC treated with pembrolizumab, two experienced prolonged progression-free survival, while one developed brain and bone metastases during therapy (24). These findings suggest that immunotherapy is effective for most patients with PD-L1-expressing PPC, although tumor heterogeneity may lead to variability in outcomes.

Additional predictive factors beyond PD-L1 expression may also influence the effectiveness of ICIs. Regarding anti-angiogenic therapy, evidence suggests that anti-angiogenic agents enhance ICI efficacy by improving the tumor microenvironment and boosting the immune response. Clinical trials have demonstrated the benefits of combining ICIs with anti-angiogenic agents in advanced NSCLC (25–27). Studies have shown that patients with PPC exhibit high levels of vascular endothelial growth factor (VEGF) (28). Oda T et al. reported that two patients with PPC were treated with carboplatin, paclitaxel, and bevacizumab, resulting in partial responses (29). Based on these findings, bevacizumab was added to this patient’s regimen, resulting in a synergistic anti-tumor effect and potentially reducing the risk of drug resistance. For bone metastases, the patient received targeted therapy with denosumab. As the first fully human monoclonal antibody, denosumab inhibits the activity of the Receptor activator of the nuclear factor-κB ligand (RANKL) and has been used to prevent bone-related adverse events (SREs) in a variety of solid tumors. Importantly, it showed synergistic anti-tumor efficacy without increased toxicity when combined with ICIs in patients with advanced non-small cell lung cancer with bone metastases (30).The patient tolerated the treatment well, with only mild bone marrow suppression and gastrointestinal side effects observed. The Common Terminology Criteria for Adverse Events (CTCAE) was created by the National Cancer Institute to classify toxicity. This patient experienced grade I leukopenia and grade I nausea, which resolved with supportive therapy.

Although the patient underwent a comprehensive genetic evaluation, and no genetic mutations were found. The epithelial and sarcomatoid components of specimens from patients with PPC were found to share genomic alterations (31). KRAS mutations are the most common in PPC, and current targeted therapies for KRAS G12C mutations show promise for NSCLC patients, further studies are still needed to investigate the therapeutic role of ras-targeted inhibitors in patients with PPC (31, 32). MET exon 14 skipping mutation (METΔex14) and epidermal growth factor receptor(EGFR) mutations are the second most common types of mutations in PPC. Capmatinib and tepotinib are approved for NSCLC patients with MET exon 14 skipping, and these drugs are expected to be used in treating PPC patients with MET exon 14 skipping (33). A patient with advanced PPC carrying an EGFR mutation with exon 19 deletion was reported to have progression-free survival up to 11 months after treatment with ositinib (34). In addition, Lin L et al. reported a case of an advanced PPC patient with a mesenchymal lymphoma kinase (ALK) rearrangement treated with crizotinib and achieved partial remission for 7 months (35). Therefore, it is particularly important to monitor genetic alterations in patients with PPC and targeted therapies against activating driver mutations may be effective in patients.

## 4 Conclusion

In summary, pleomorphic carcinoma of the lung is a highly aggressive tumor with a high risk of rapid recurrence, even in stage IA1 disease treated with surgery alone. The role of adjuvant chemotherapy, radiotherapy, and immunotherapy requires further investigation. As a sarcomatoid carcinoma containing spindle cell and/or giant cell components, PPC responds well to gemcitabine combined with paclitaxel. For patients with high PD-L1 expression, the use of PD-1/PD-L1 inhibitors is essential. In this case, the combination of chemotherapy, immunotherapy, and anti-angiogenic therapy effectively controlled the disease, leading to significant tumor shrinkage. The patient continues to receive maintenance therapy with combined immunotherapy and anti-angiogenic therapy to prolong survival.

## Data Availability

The original contributions presented in the study are included in the article/supplementary material. Further inquiries can be directed to the corresponding authors.
